# Incipient Cognition Solves the Spatial Reciprocity Conundrum of Cooperation

**DOI:** 10.1371/journal.pone.0017939

**Published:** 2011-03-15

**Authors:** Jeromos Vukov, Francisco C. Santos, Jorge M. Pacheco

**Affiliations:** 1 Applications of Theoretical Physics Group, Centro de Matemática e Aplicações Fundamentais, Complexo Interdisciplinar da Universidade de Lisboa, Lisboa, Portugal; 2 Departamento de Informática & Centre for Artificial Intelligence, Universidade Nova de Lisboa, Caparica, Portugal; 3 Departamento de Matemática e Aplicações, Universidade do Minho, Braga, Portugal; Cedars-Sinai Medical Center and University of California Los Angeles, United States of America

## Abstract

**Background:**

From the simplest living organisms to human societies, cooperation among individuals emerges as a paradox difficult to explain and describe mathematically, although very often observed in reality. Evolutionary game theory offers an excellent toolbar to investigate this issue. Spatial structure has been one of the first mechanisms promoting cooperation; however, alone it only opens a narrow window of viability.

**Methodology/Principal Findings:**

Here we equip individuals with incipient cognitive abilities, and investigate the evolution of cooperation in a spatial world where retaliation, forgiveness, treason and mutualism may coexist, as individuals engage in Prisoner's Dilemma games. In the model, individuals are able to distinguish their partners and act towards them based on previous interactions. We show how the simplest level of cognition, alone, can lead to the emergence of cooperation.

**Conclusions/Significance:**

Despite the incipient nature of the individuals' cognitive abilities, cooperation emerges for unprecedented values of the temptation to cheat, being also robust to invasion by cheaters, errors in decision making and inaccuracy of imitation, features akin to many species, including humans.

## Introduction

Undoubtedly one of the most important legacies of Biology to Mathematics, Evolutionary Game Theory (**EGT**) [1], [2] has been widely employed in the study of the evolution of cooperation, spanning a plethora of research areas which investigate this fascinating problem. **EGT** introduces a population dynamical view of Game Theory, in which one no longer needs to invoke any rational behaviour of individuals [3]. Hence, the evolution of cooperation can be investigated in populations of arbitrary constituents. Recently, instances of the Prisoner's Dilemma (**PD**) game have been identified in simple organisms such as phages and bacteria [4]–[6].

In the one-shot **PD**, each interaction involves two persons, who can act as Cooperators (***C***) or Defectors (***D***). A ***C*** is one who contributes a cost *c* to confer to the other a (larger) benefit *b*; otherwise she is a Defector (***D***). Hence mutual cooperation confers a net positive benefit *b−c*, whereas mutual defection confers nothing to both players. Cooperating towards a ***D*** means to pay a cost without receiving any benefit, hence ending up with a payoff of *−c*, whereas the ***D*** gets *b*, as she accesses the benefit at no cost. Under the conventional assumptions of **EGT** – infinite well mixed populations – cooperators always fare worse than defectors, and natural selection will favour the latter. However, when populations are spread in space and individuals can only interact with their neighbours, cooperation may become evolutionary viable, as beautifully illustrated by Nowak and May back in 1992 [7], making use of a simplified version of the **PD**. This so-called spatial reciprocity mechanism relies on the fact that unconditional players have a limited set of fixed neighbours to interact with that allows ***C***s to protect themselves from ***D***s by self-organizing into compact clusters, thereby minimizing the risk of exploitation by cheaters [7]–[9] (***D***).

**Figure 1:**
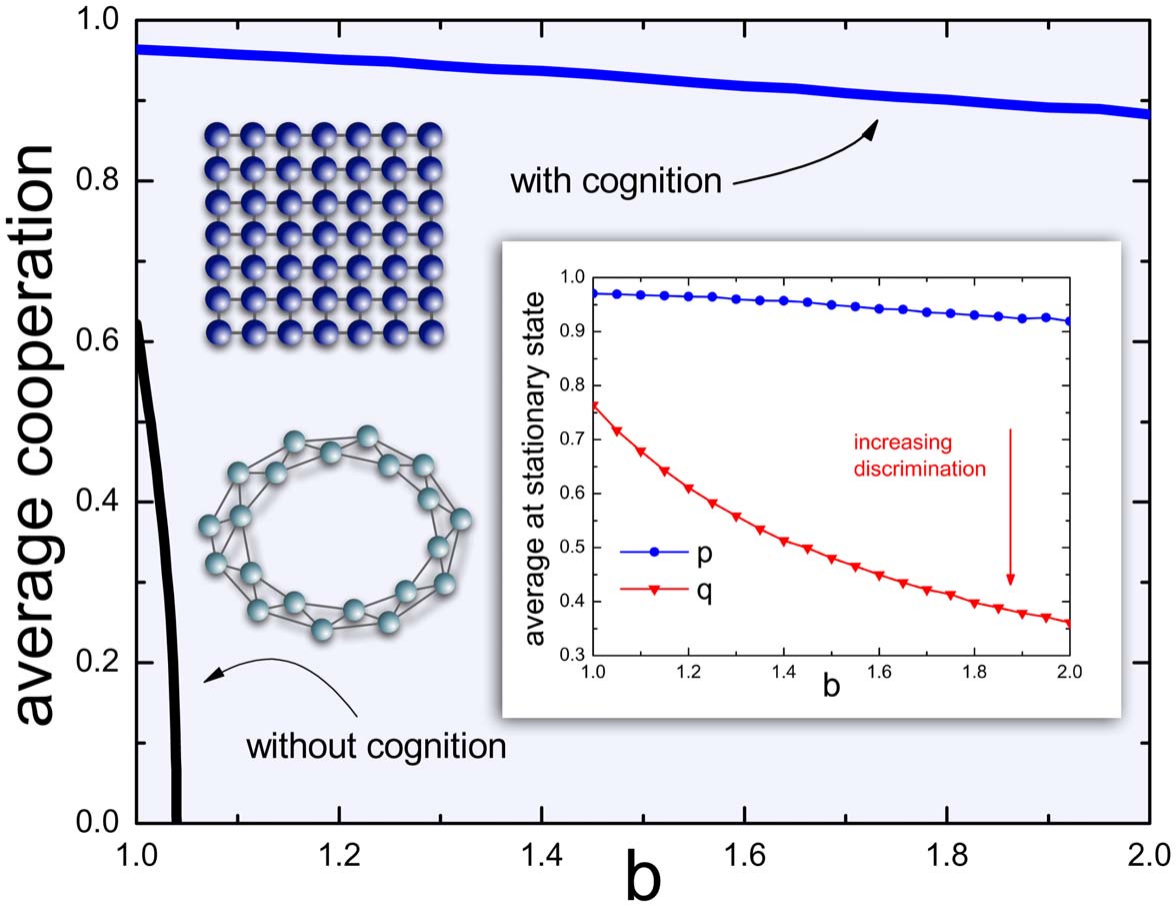
Cooperation and Cognition under spatial reciprocity. When compared to the conventional result for unconditional strategies under spatial reciprocity (black line), inclusion of incipient cognitive abilities makes cooperation dominant even when temptation to cheat *b* is high (blue line). In such cases, cooperators adopt increasingly retaliatory decisions against cheaters (*q* value decreases with increasing *b*; see main text for details).

Spatial reciprocity, however, provides a rather narrow window of opportunity for cooperators to evolve under the **PD**, as illustrated with the black solid line in Figure 1 (simulation methods for the unconditional strategies are detailed in the Supporting Information, File S1). While this result has prompted the search for other mechanisms that may favour the emergence of cooperation, nowhere was it taken into account that, in many species, it will be almost impossible to imagine players to adopt an immutable, unconditional behaviour towards all their neighbours, however few.

Recent progresses in identifying neural correlates of behaviour, not only in Humans [10], [11] or the upper primates, but also in other species [12], prompt one to abandon one of the pillars of **EGT**: the lack of cognition of the population constituents. Introducing (social) cognition [13], [14] into **EGT**, however, opens up a plethora of possibilities, very much like letting individuals engage in repeated interactions with each other [15]. Here we shall equip individuals with the simplest form of social cognitive ability. As a result, we obtain the solid blue line in Figure 1. As soon as individuals exhibit incipient cognitive capacities, cooperation blooms. In the following we show how this happens, proving further that the result is extremely robust to errors, and that cooperating populations are able to withstand fierce invasion attempts from cheaters.

## Results and Discussion

The lattice depicted in Figure 1 (upper graph) illustrates the conventional population layout under spatial reciprocity, in which individuals are located in two-dimensional space, occupying the nodes of the lattice, interacting with those individuals they are linked to. Spatial lattices constitute examples of regular graphs, and our results apply qualitatively to any graph in which every individual has the same number of neighbours (e.g. Figure 1, lower graph). As a result, the only parameter characterizing such a graph is the number of neighbours of each individual, which we denote by *k*. Moreover, we shall further normalize the reward for mutual cooperation making *b−c* = 1, thereby reducing the **PD** to a one-parameter game with *b*>1 [16].

**Figure 2:**
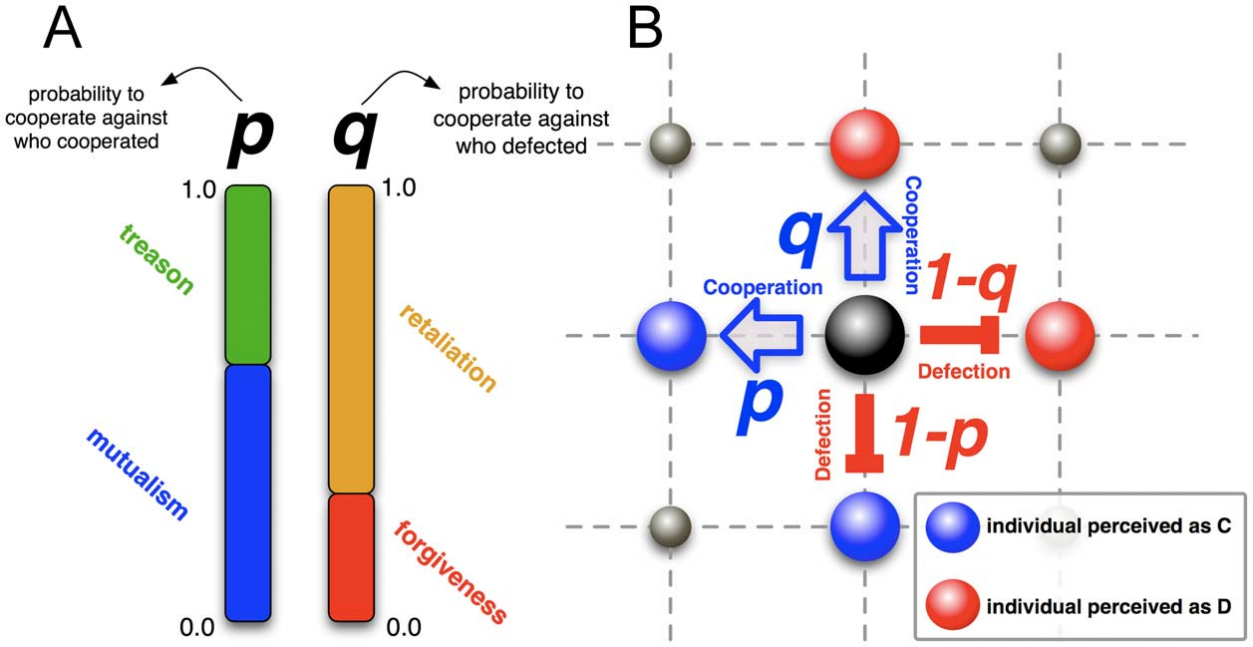
Modelling individual cognition. **A**) In each interaction, individuals choose simultaneously between two possible actions: to cooperate or to defect. This choice follows a stochastic decision process characterized by two parameters *p* and *q*. While *q* allows individuals to retaliate or to forgive a bad action, *p* defines the probability to reward a good action received in the past with another one. **B**) Each individual (*p*,*q*) values define how she behaves towards her neighbours as interactions proceed along the links of a spatial lattice (regular graph), allowing individuals to adopt different actions depending on what each neighbour did in the past.

Let us consider myopic individuals whose only information they manage is that resulting from their interactions. Equipping these individuals with cognitive abilities will let them discern cooperative from defective actions towards them, and perhaps react differently to those actions. Since, whenever two individuals interact, they make a simultaneous decision of what to do, then at the simplest level, information available will correspond to the last time the two individuals have interacted, a feature which empirical studies suggest as reasonable [17], [18]. Clearly, this is the simplest possible level of cognition, which we denote by incipient cognition, as opposed to other, more elaborate forms of cognition [12], [19]. Because decision making is not a deterministic process [14], [20], we associate each individual interaction with a stochastic decision process characterized by two parameters *p* and *q*. As illustrated in Figure 2B, a (*p,q*) strategist will cooperate (defect) against a neighbour with probability *p* (*1−p*) if the given neighbour cooperated with her in their previous interaction. Similarly she cooperates (defects) with probability *q* (*1−q*) if the neighbour defected against her in the previous interaction. A similar model was studied by Nowak and Sigmund in the framework of the two-player iterated PD and well-mixed populations [21]–[26]. The parameter *p* can be understood as a measure of mutualism and (*1−p*) as a propensity for treason. Similarly, *q* provides a qualitative measure of forgiveness, whereas (*1−q*) measures the individual tendency to retaliate, as illustrated in Figure 2A. Unconditional strategies correspond in this framework to extreme cases: Unconditional cooperation to (*1,1*) and unconditional defection to (*0,0*). It is worth noting that retaliatory strategies, such as (*1,0*), resemble the famous ‘Tit for Tat’ strategy so popular in the context of the iterated **PD** [27].

The description above means that the players have (short term) memory about what others did to them previously and can use this knowledge in the process of decision making. Another important feature of stochastic decision making is that players can take different actions (to cooperate or to defect) against different neighbours, even if the neighbours acted identically in the previous interaction. Successful individuals will be imitated by their peers, with successful (*p,q*) pairs spreading through the population. In addition, every imitation process entails inherent errors of *decision* and *perception* when strategies are assessed and eventually copied. Errors of perception have potential relevance given that individuals do not have direct access to the set of rules that define the behaviour of their neighbours, but to their actions. Consequently, the imitation process naturally contains some level of inaccuracy.

The results shown in Figure 1 (solid blue line) correspond to populations of size *N* = 10000 and *k* = 4, which have evolved as described above and in Materials and Methods, starting from players with (*p,q*) strategies (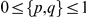) drawn randomly from a uniform distribution.

Figure 1 shows the fraction of cooperative actions in the population as a function of the temptation to defect *b*. Analysis of the average values of *p* and *q*, in the stationary state, illustrated in the inset, also provides interesting information. Individuals willing to cooperate can invade the whole population by quickly creating cooperative clusters, which allows them *i*) to profit from mutual cooperative acts and *ii*) to defend themselves from exploiters as they adopt low values of *q*. This leads to the fixation of individuals with a high *p* value almost independently of the measure of the temptation (*b*). Players with high *p* values at both ends of a link leads to a stable cooperative link because they will most likely cooperate subsequently after a mutual cooperative act. Thus, high average *p* values constitute a good indicator of high overall cooperation in the population. Global mutual cooperation is only set back due to occasional defection as a result of the stochastic nature of the decision making and of imperfect behavioural copying. In what concerns the behaviour of the average *q*-value, we observe that for low temptation *b* (and low value for the cost *c*) the stationary *q*-value is rather high as an occasional defection does not cause a big loss to the cooperative partner and fast forgiveness pays off. In other words, mild dilemmas bring along weak selection towards retaliation. On the contrary, for larger values of *b* (and *c*), selection for more retaliatory behaviour increases, and low *q*-values become more advantageous. These results show a lower propensity for forgiveness than it was found in the well-mixed case [23], [28], which can be explained by the fixed connections and harder retaliation towards neighbours. It is noteworthy that, whenever the dilemma is strict (high *b*), the emergent retaliative strategies enclose the same principles as the most successful norm in promoting cooperation in the framework of indirect reciprocity, where stern punishment against defectors is compensated by prompt forgiving each time a defector turns into a cooperator [29].

Let us now investigate the robustness of cooperation to cheater invasion. To this end we replaced, in every generation, a given fraction of players (randomly chosen) with (*0,0*) strategists. The results (for details, see File S1), show that cooperative strategies persist even in the most adverse conditions (highest *b*); the prompt reaction of players in isolating defectors renders the fitness of the intruders far below that of their ‘cooperator’ neighbours. Hence, defector invaders quickly resume to cooperation. It is worth to mention that these results were obtained under an evolutionary timescale in which individuals revise their strategy after every interaction. This makes it harder for cooperators to identify freshly “injected” defectors and retaliate against free-riders, even if, as we show, cooperation remains robust in this setting. Investigating the effects of the different timescales on the evolution of the strategies when the system starts from a random initial condition, i.e., not from an established cooperative environment, is a more difficult problem. It is well known that the number of game rounds before each strategy revision may play an important role [30]–[32]. In this sense, our model may foster further studies concerning this issue.

**Figure 3:**
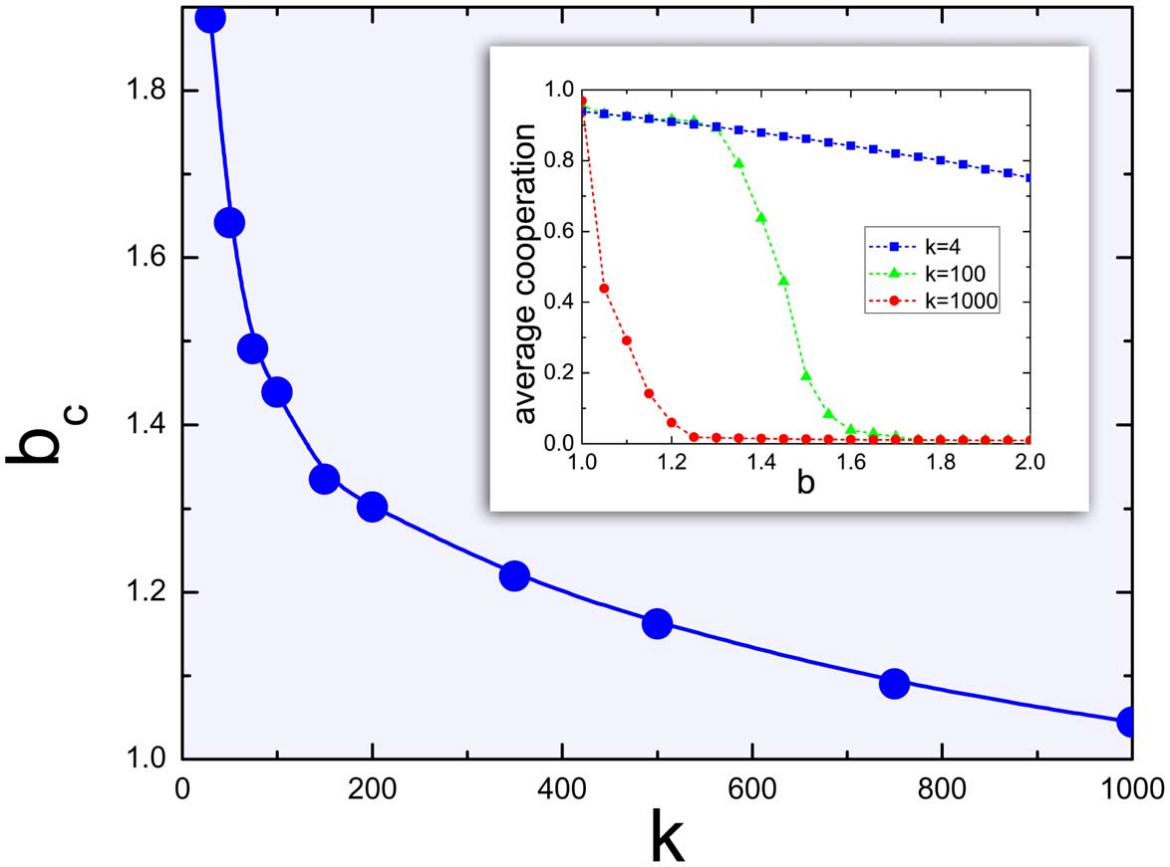
Robustness of cooperation with increasing neighbourhood size *k*. Main panel: We plot the temptation values *b_c_* below which the fraction of cooperative actions becomes higher than 50% in the population, as a function of neighbourhood size *k*. Inset: We plot the average fraction of cooperative actions as a function of the temptation to defect *b* for different values of connectivity *k*. Incipient cognitive abilities help individuals to establish and maintain cooperation even for a very high number of neighbours. The simulations were carried out in a population of 10.000 individuals placed on regular ring graphs (lower graph in Figure 1).

Finally, in Figure 3 we show how the emergence of cooperation among incipient cognitive agents remains possible even when the number of neighbours increases substantially. Naturally, with increasing *k*, cooperation becomes harder to emerge, although its demise is slow and progressive, in sharp contrast with what has been observed with unconditional players [16]. In connection with Figure 1, Figure 3 also uncovers a detailed interplay between cognition and the size of the social cliques below which cooperation remains stable, which may have an impact in the evolution of cognition and the social brain hypothesis [33], [34].

Our results provide strong evidence that cognition, even at its most incipient level, obliterates the advantage of defectors in spatial dilemmas of cooperation. They also prompt one to combine population dynamics with different cognitive mechanisms to unveil the complex and diverse features of animal cooperation. Even in the absence of repeated interactions, reputation or punishment, incipient cognition makes spatial cooperation evolutionary viable throughout most of the parameter space of interest for the prisoner's dilemma. Hence, the role of cognition in the evolution of cooperation should not be overlooked, being it cast in terms of two-person interactions, or in terms of group interactions.

## Materials and Methods

Players are located on the nodes of a graph. The edges of the graph define who interacts/imitates who. Individuals engage in single-shot **PD** games with each of their *k* neighbours in each simulation step and gain the accumulated payoff from these interactions. Each has to make *k* decisions contingent on her own *p* and *q* parameters and the particular actions of her neighbours the last time they interacted. Computer simulations start from a population where individuals are assigned random values of *p* and *q*, drawn from a discretized strategy space with values p = i*0.01 and q = j*0.01 (*i,j = 0,…, 100*). Given the lack of information at start, every individual cooperates with probability *(p+q)/2* or defects with probability [*1−(p+q)/2*]. In each simulation time step, we randomly pick two neighbouring players (*x* and *y*), and calculate their individual payoff (fitness); player *x* adopts the strategy of player *y* with a probability given by 
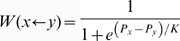
, corresponding to the so-called pairwise comparison rule [35]. *P_x_* and *P_y_* are the individual fitness of players *x* and *y* while *K* is associated with errors in decision making. In addition, whenever a player decides to adopt the strategy of her neighbour, the new strategy parameters will be *p_x_′ = p_y_+ξ_1_(σ)* and *q_x_′ = q_y_+ξ_2_(σ)*, where *ξ_1_(σ)* and *ξ_2_(σ)* are normally distributed random variables with zero mean and standard deviation of *σ*. This feature can model a slight blur in perception and helps to avoid the random extinction of strategies; it also ensures a complete exploration of the strategy spectrum, given that the pairwise comparison does not introduce new strategies in the population.

Results in Figure 1 were obtained performing extensive computer simulations on a square lattice (*N = 100×100*, illustrated in dark blue in the inset of the same figure), employing the so-called von Neumann (also known as Manhattan) neighbourhood (*k* = 4). We imposed periodic boundary conditions and let the system evolve for 10000 generations. Subsequently, we averaged the particular strategy concentrations over the population during additional 100000 generations. The *K* parameter of the strategy update was chosen to be 0.4 as this value was proven to be favourable for cooperation in the case of unconditional strategies [36] (black curve in Figure 1). The standard deviation *σ* associated with errors in imitation was taken to be *σ = 0.005*. Simulations for Figure 3 were executed on ring-graphs of the size N = 10000 and varying connectivity *k*. The equilibrium average *p* and *q* values were obtained from averaging over 10000 generations after a transient period of 5000 generations for 100 different random initial conditions. The results are also independent of the type of updating (synchronous versus asynchronous).

## Supporting Information

File S1 **Supporting information.**
(PDF)
