# Supplementary material for: Incipient Cognition Solves the Spatial Reciprocity Conundrum of Cooperation
Source: PLoS One. 2011 Mar 15;6(3):e17939. doi: 10.1371/journal.pone.0017939 (PMC3058056; doi:10.1371/journal.pone.0017939)
Supplement: File S1 — Supporting information. (PDF) [file pone.0017939.s001.pdf]

SUPPORTING INFORMATION

Incipient Cognition Solves the Spatial Reciprocity Conundrum of Cooperation

Jeromos Vukov[1], Francisco C. Santos[2,1], Jorge M. Pacheco[1,3]

[1] *ATP-group, Centro de Matemática e Aplicações Fundamentais, Complexo Interdisciplinar da Universidade de Lisboa, 1649-003 Lisboa-Codex, Portugal.*

[2] *CENTRIA, Universidade Nova de Lisboa, Quinta da Torre, 2829-516 Caparica, Portugal*

[3] *Departamento de Matemática e Aplicações, Universidade do Minho, 4710-057 Braga, Portugal*

Simulations using unconditional strategies (Figure 1)

The simulations for the solid black curve in Figure 1 were executed on a square lattice of the size *N=200x200* with periodic boundary conditions. Players located on the nodes of the square lattice could follow the C (always cooperate) or D (always defect) strategies. We used random initial conditions where both strategies were present with the same frequency. Players gained their payoff from Prisoner's Dilemma Games with their four nearest neighbours and had the opportunity to imitate the strategy of one of their (randomly chosen) neighbours with the probability $W(x \leftarrow y) = \frac{1}{1+e^{(P_x - P_y)/K}}$, where x stands for the focal player and y for the possibly imitated partner. P_x and P_y are the total payoffs of the players, respectively, while K characterizes the noise in decision making. We let the system evolve for 20000 generations then averaged the strategy concentrations over the population for additional 50000 generations. We chose the $K=0.4$ parameter value because this value is close to optimal for cooperation [1].

Robustness against defector invasion

To investigate how cooperation is robust against invasion by defectors, we started the simulations from random initial strategy values p and q and, after a transient time (2000 generations), randomly replaced a given fraction μ of players by ($0,0$) strategists in every generation (that is, N elementary steps). Figure S1 shows the average p and q values for different temptation parameters and defector invasion rates. High p values illustrate that cooperation persists even for the highest temptation *and* for considerably high invasion

rates. The incipient cognitive abilities of players enable them to react promptly and isolate defectors by decreasing their fitness below that of the 'cooperator' neighbours. As a consequence, the invaders are quickly 'converted' back to cooperators. In order to give defectors a chance to invade the population, it is necessary to artificially increase the injection rate of pure defectors to values which effectively counteract the characteristic time scale of defector-to-cooperator conversion.

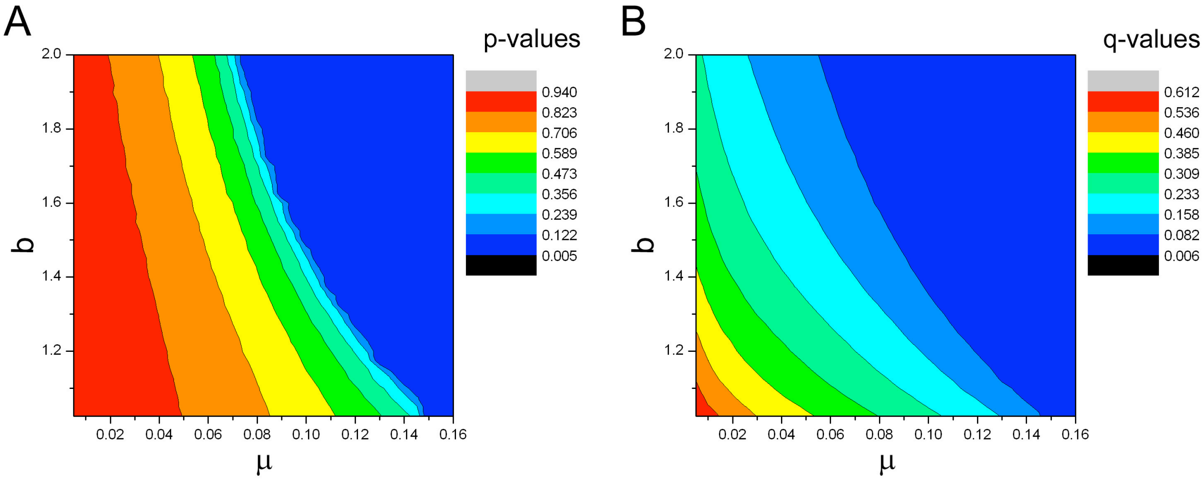

Figure S1. **Robustness of cooperation against defector invasion.** Final average p and q values as a function of temptation (b) and percentage of injected pure defectors (μ). High p values indicate high overall cooperation. Cooperation is stable against defector invasion even for the biggest b parameter values. The results correspond to simulations carried out on a square lattice of size N=100x100.

Strategy-parameter distribution for different temptations

The inset in Figure 1 provides information on the average stationary p and q values.

However, their distribution is also worth investigating. Contrary to the distribution of p-values that is sharply concentrated at one, and remains so for a wide range of values of b, the high fraction of cooperative actions promotes a significant dispersion of q-values for all types of individuals, as it creates a smaller selection pressure for the evolution of this particular trait. In what concerns the average p and q values, the inset of Figure 1 shows that, with increasing b, the dominating strategies approach maximum discrimination ($1,0$): High temptation values leave no room for tolerance and cooperators survive based on sharp discrimination. Moreover, as discrimination increases, the dispersion in q is also reduced: we obtain a 13% reduction in q-dispersion as b increases from 1.2 to 2.0. This situation is similar to that found in direct reciprocity contests, where sharp discrimination and prompt forgiving (TFT) was the winning strategy against a diversity of tournament strategists.

Changing the copying accuracy (σ) influences the distributions accordingly, as the dispersion of both p and q increases with increasing σ. There is however another side-effect: Because $0 \leq p,q \leq 1$, strategy parameters falling outside this interval (and arising from copying with accuracy σ) must be discarded. As a result, the peak at $p=1$ can be shifted to slightly lower values if σ is increased, as the total probability of acquiring a lower p value while imitating a $p=1$ player becomes larger than the probability of perfect imitation.

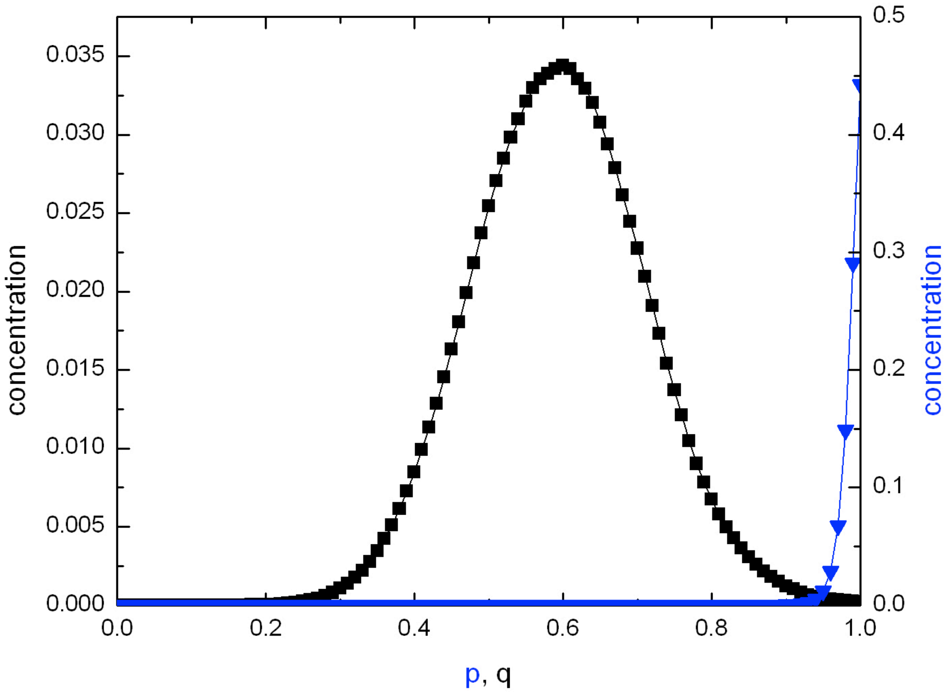

Figure S2: Strategy distribution on square lattice for *b*=1.2 and *σ*=0.0025. Blue triangles (black squares) show the distribution of *p* (*q*) values. The right (left) scale applies to the *p* (*q*) distribution. While the distribution of *p* rapidly converges to a sharp function peaked at one, *q* evolves under low selection pressure, being able to maintain appreciable levels of diversity.

REFERENCES

1. Szabó G, Vukov J, Szolnoki A (2005) Phase diagrams for an evolutionary prisoner's dilemma game on two-dimensional lattices. Phys Rev E 72: 047107.
